# Cooperative Motion Optimization Based on Risk Degree under Automatic Driving Environment

**DOI:** 10.3390/s24134275

**Published:** 2024-07-01

**Authors:** Miaomiao Liu, Mingyue Zhu, Minkun Yao, Pengrui Li, Renjing Tang, Hui Deng

**Affiliations:** 1 School of Transportation Science and Engineering, Beihang University, Beijing 100191, China; zmymoon@buaa.edu.cn (M.Z.); 20374127@buaa.edu.cn (M.Y.); 19374145@buaa.edu.cn (P.L.); renjingtang@buaa.edu.cn (R.T.); 2 China Telecom System Integration Co., Ltd., Baoding 071700, China; zmybuaa@126.com

**Keywords:** cooperative strategy, motion optimization, autonomous vehicles, risk degree, traffic simulation

## Abstract

Appropriate traffic cooperation at intersections plays a crucial part in modern intelligent transportation systems. To enhance traffic efficiency at intersections, this paper establishes a cooperative motion optimization strategy that adjusts the trajectories of autonomous vehicles (AVs) based on risk degree. Initially, AVs are presumed to select any exit lanes, thereby optimizing spatial resources. Trajectories are generated for each possible lane. Subsequently, a motion optimization algorithm predicated on risk degree is introduced, which takes into account the trajectories and motion states of AVs. The risk degree serves to prevent collisions between conflicting AVs. A cooperative motion optimization strategy is then formulated, incorporating car-following behavior, traffic signals, and conflict resolution as constraints. Specifically, the movement of all vehicles at the intersection is modified to achieve safer and more efficient traffic flow. The strategy is validated through a simulation using SUMO. The results indicate a 20.51% and 11.59% improvement in traffic efficiency in two typical scenarios when compared to a First-Come-First-Serve approach. Moreover, numerical experiments reveal significant enhancements in the stability of optimized AV acceleration.

## 1. Introduction

With the widespread application of intelligent and connected technologies in the transportation domain, the conventional intersection organization model has become obsolete. To optimize the utilization of spatiotemporal resources at intersections and enhance traffic efficiency, harnessing the advantages of autonomous vehicles (AVs) and embracing an innovative cooperative motion optimization strategy is crucial.

To bolster intersection efficiency, a pivotal approach involves the proposition of novel traffic regulations. Dresner et al. [1] introduced the concept of autonomous intersection management (AIM), which relies on a reservation system to supplant the traditional signal-based scheme with real-time phase adjustments driven by dynamic AV requests. The AIM method operates on the principles of First-Come-First-Served (FCFS) and conflict-free criteria. Li et al. [2] assessed the efficacy of the AIM method using VISSIM. Nevertheless, Levin et al. [3] noted that the AIM method exhibits less effectiveness than the traditional signal scheme in certain scenarios, especially when confronted with high traffic demand. Cui et al. [4] investigated the problem of optimally allocating input rates to entry links and simultaneously finding a stabilizing signal control policy with phase fairness.

Subsequent studies have explored the optimization of AV traffic patterns, incorporating deep reinforcement learning algorithms [5] and intersection coordination algorithms with discrete time-occupied trajectories [6]. These endeavors yielded substantial improvements compared to FCFS, which are explored in greater depth. AV sequencing was optimized using mixed-integer linear programming [7], and virtual AV mapping techniques [8] were employed. Mieheli et al. [9] developed a dynamic programming model focused on maximizing intersection throughput. The model’s solution leveraged the Monte Carlo tree search algorithm to determine optimal accelerations for conflict prevention. Zhu et al. [10] proposed a dynamic coordinated control method utilizing linear programming. This approach introduced a dual-level, lane-based optimization model based on an optimized grid system, considering time-varying departure times and trajectory selection to facilitate traffic flow propagation in the network. Zhuo et al. [11] investigated the effect of CAV platoon configurations at a typical isolated roundabout in a mixed traffic environment to enhance the efficiency. However, it is worth noting that these studies primarily simplified scenarios to single-lane conditions.

The core of the efforts to enhance intersection traffic efficiency revolves around controlling AV motion parameters. These studies mainly govern the duration of AV presence at intersections or employ monitoring hardware for precise AV control. A well-recognized strategy involves dynamically adjusting the arrival times of AVs at intersections. This is achieved by solving the optimal control problem for AVs under state constraints using the direct adjacency method [12], with the aim of achieving high efficiency and minimal energy consumption. On the hardware front, real-time information on AV position and speed can be obtained through connected technologies and multi-sensor integration methods [13,14], facilitating effective collision avoidance. Models for AV-aware determinations, such as those based on dynamic Bayesian networks [15], can calculate confidence levels for acceleration and deceleration based on AV collision probabilities, providing guidance for traffic decisions. Xue et al. [16] designed an observer-based event-triggered adaptive platooning control algorithm for autonomous vehicles (AVs) with motion uncertainties (e.g., unknown AV mass, internal resistance, and external disturbances).

Managing potential conflicts among AVs assumes primary importance in ensuring safety. Previous research has generally defined conflict as the intersection of trajectories between two vehicles, with varying degrees of severity. Unacceptable conflict results in collisions, making the discrimination of conflict severity a crucial measure of collision likelihood. Numerous studies have determined conflict severity by establishing thresholds for vehicle motion parameters, classifying these parameters into five categories: the presence of conflict avoidance behaviors, the proximity of AVs in terms of time and space, the motion characteristics of the traffic subjects, the destructive energy associated with a potential collision, and other integrated metrics [17].

The category of the “presence of conflict avoidance behaviors” pertains to significant slowing or swerving actions exhibited by conflicting AVs [18]. However, this method is inherently subjective, prone to biased definitions and observations, and lacks essential indicators, such as speed and distance. In contrast, the “proximity of AVs in time or space” considers parameters such as distance, speed, and time. Notable numerical indicators in this category include the time to collision (TTC) [19] and post-encroachment time (PET) [20], both inversely correlated with conflict severity. Severe collisions occur when TTC and PET values drop below predefined thresholds. Studies focusing on the traffic subject’s motion characteristics often utilize the deceleration rate (DR) as an indicator to avoid crashes. According to the research in [21], DR values are positively correlated with conflict severity. Nevertheless, it is important to note that the thresholds for the TTC, PET, and DR are subject to artificial definitions.

Research that employs conflict energy as an indicator considers both conflict severity and the potential for collisions [22]. However, such studies require substantial data support, posing significant challenges. In the domain of integrated indicators, researchers have utilized microscopic motion parameters to discern conflict behavior. For instance, Liu et al. [23] represented AVs as circles with their center of mass as the circle’s center. They determined conflict behavior through video processing algorithms, conflict location detection, and conflict time thresholds. While this approach aligns more closely with reality, it demands extensive data and detailed motion parameters.

The overarching objective of this paper is to enhance the overall intersection efficiency by optimizing AV trajectories and movements within intersections. AVs have the flexibility to select appropriate exit lanes, thereby maximizing the utilization of intersection space resources. Subsequently, a motion optimization method grounded in risk assessment is devised to regulate microscopic AV motion parameters, enabling conflict avoidance and efficiency enhancements. This work facilitates the efficient utilization of intersection time resources. Ultimately, an all-inclusive cooperative motion optimization model is developed, extending the study to include the entirety of AV interactions within intersections. Numerical and simulation experiments validate the efficacy of this paper’s methodology, underscoring the effectiveness of the cooperative motion optimization strategy in improving efficiency.

The contributions of this paper to the existing body of literature can be summarized as follows:

1. This paper effectively utilizes intersection space resources. While numerous studies have explored the optimization of AV trajectories, many of them have oversimplified scenarios by focusing on single-lane situations, which do not fully reflect the real demands of AVs. To address this gap in the research, this paper allows for AVs to freely select lanes corresponding to their intended exits.
2. This paper introduces a linear control mechanism for the acceleration and speed of AVs, grounded in the cooperative motion optimization strategy. This approach dynamically adjusts AV movement based on their current states, ensuring that they can traverse intersections safely and at an optimal speed.
3. While previous research has made strides in determining conflict severity, many of these methods still rely on subjective threshold judgments, lacking a uniform and objective criterion for conflict assessment. To address this issue, this paper formulates a risk degree to assess conflict severity, relying solely on numerical comparisons of objective parameters.

The remainder of this paper is structured as follows:

Section 2 elaborates on the method used for selecting AV trajectories. Section 3 provides an in-depth explanation of the formulation of the motion optimization method based on the concept of risk degree. Section 4 explores the cooperative motion optimization strategy for AVs. In Section 5, numerical and simulation case studies conducted at signalized intersections are presented to illustrate the effectiveness of the proposed cooperative strategy. Finally, the last section concludes this paper, offering discussions on the findings and outlining potential directions for future research.

## 2. Trajectory Selection Formulation

**Figure 1:**
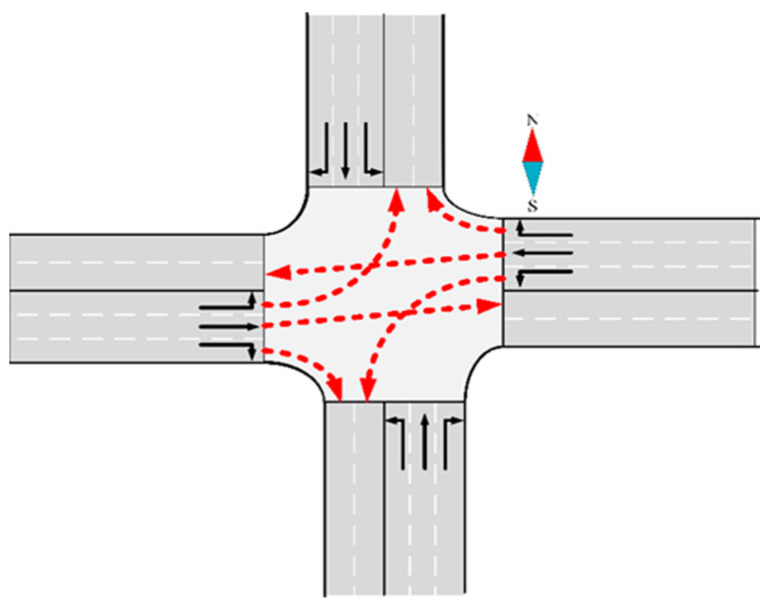
Common intersection vehicle trajectory assumptions.

AVs are limited to one exit lane in most studies. Figure 1 illustrates the scheme of the trajectory selection in previous papers.

**Figure 2:**
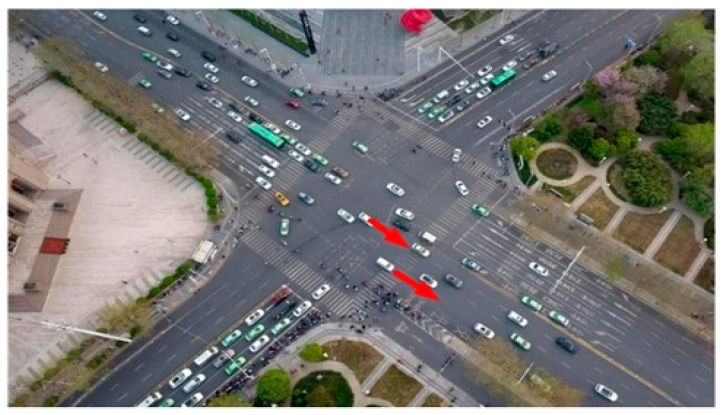
Zhengzhou Garden Road Dongfeng intersection.

However, in real life, vehicles can actually take any lane, such as the Zhengzhou Garden Road Dongfeng intersection shown in Figure 2.

**Figure 3:**
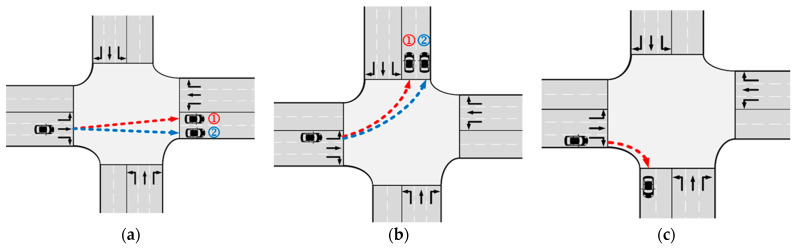
The assumptions of the trajectory selection method in this paper. (**a**) The trajectory selection of the through vehicles; (**b**) the trajectory selection of the left-turn vehicle; and (**c**) the trajectory selection of the right-turning vehicle.

Hence, in this paper, AVs have the flexibility to select any lanes within their corresponding exits, thereby expanding the available space resources. The specific assumptions are outlined as follows:

1. The AVs depicted in Figure 3a,b, which include both through and left-turn AVs, are permitted to choose lanes within the exit without restrictions.
2. However, the right-turning AVs illustrated in Figure 3c are constrained to utilize only the outer lane within the exit. Because right-turning vehicles are generally not constrained in Chinese traffic rules, we adopt by default that right-turning vehicles are not free to choose their own lanes to reduce conflicts. This restriction is imposed to minimize potential conflicts.

Building upon the trajectory selection method for AVs as outlined earlier, Section 3 presents the formulation of a motion control method grounded in the concept of risk degree. Simultaneously, this motion control method has the capability to determine the optimal lane choice with a focus on enhancing efficiency.

## 3. Motion Optimization Formulation

This section provides an overview of the foundational framework for motion optimization, a central focus of this paper. The Avs’ motion parameters are subject to analysis and linear control, guided by the trajectories generated in Section 2. The result of this optimization process is the determination of a total traveling time indicator, which serves as the basis for selecting an optimal route.

### 3.1. Risk Degree

Commonly used risk indicators such as the TTC, PET, DRAC, etc., often require statistical and subjective judgement of a threshold value to define the degree of danger through measured data, which will have a certain degree of human subjective error and cannot take advantage of the potentially useful time of the conflict to a large extent. Therefore, we choose to use an objective indicator, i.e., risk degree. The risk degree serves as a more objective indicator for determining conflict severity, relying on objective parameter comparisons. Two primary objective parameters are utilized in this context: the post-encroachment time (PET) and conflict point occupancy time (CPOT). The PET quantifies the time interval needed for AVs to reach the conflict points, while the CPOT denotes the time that the conflict points can accommodate the passage of the next AV. These parameters consider the AV speed, distance, and time, facilitating an objective comparison. Consequently, the risk degree eliminates the need for subjective judgment in the conflict severity assessment.

#### 3.1.1. Conflict Points

**Figure 4:**
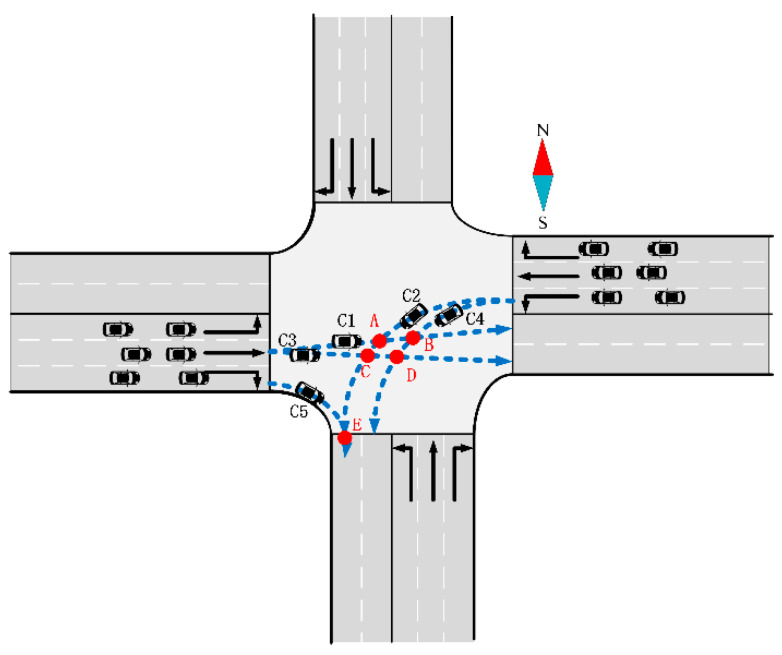
Conflict points A, B, C, D, and E.

The simplified map of intersections used in this paper is simplified from a real-life intersection with three inlet lanes and two exit lanes in each direction. A conflict point represents a juncture where two traffic trajectory lines intersect within an intersection. These conflict points are categorized into two types: merging and crossing conflict points, as illustrated in Figure 4.

#### 3.1.2. Post-Encroachment Time

The PET metric has found widespread application in the a posteriori analysis of traffic data, as documented in previous studies [20,24]. The PET quantifies the time gap between one actor departing from and another actor entering a specified conflict area. Assuming that AV *C*_1_ precedes AV *C*_2_ in passing conflict point A, we define the PET as expressed in Equation (1):

(1) $$PET\left(C_{1},C_{2},A\right)=t_{\mathrm{entry}\;}\left(C_{2},A\right)-t_{\mathrm{exit}\;}\left(C_{1},A\right)$$

The PET predominantly encapsulates the influence of the AVs’ speed and time on their safety, as the effect of other intersection attributes (e.g., sight distance, slope, etc.) on predictive accuracy is minimal.

The PET is expressed as *t*.

#### 3.1.3. Conflict Point Occupancy Time

The conflict point occupancy state signifies the phase during which the AV’s entire body has successfully traversed the conflict point. The CPOT, on the other hand, represents the minimal duration from the initiation of conflict point occupancy until the subsequent AV is permitted to approach. The CPOT is mathematically represented as $t_{c}$, whose value demonstrates a negative correlation with the probability of a collision occurring.

For the crossing-conflict scenario in Figure 4, if $C_{1}$ firstly occupies conflict point *A* and the waiting AV is $C_{2}$, $t_{c}$ can be expressed as Equation (2):

(2) $$t_{c}=\frac{l_{c1}+{0.5w}_{c2}}{v_{c1}}$$

If $C_{2}$ firstly occupies conflict point *A* and the waiting AV is $C_{1}$, $t_{c}$ can be expressed as Equation (3):

(3) $$t_{c}=\frac{l_{c2}+{0.5w}_{c1}}{v_{c2}}$$

where $v_{c1}$ is the speed of $C_{1}$, $v_{c2}$ is the speed of $C_{2}$, $l_{c1}$ is the length of $C_{1}$, $l_{c2}$ is the length of $C_{2}$, $w_{c1}$ is the width of $C_{1}$, and $w_{c2}$ is the width of $C_{2}$.

For the merging-conflict scenario in Figure 4, the AV speed difference is included, as passing the conflict point may cause a prolonged occupation time. In this scenario, the left-turn AV is $C_{2}$ and the right-turn AV is $C_{5}$.

If $C_{2}$ firstly occupies conflict point *A*, $t_{c}$ can be expressed as Equation (4):

(4) $$t_{c}=\frac{l_{c2}}{v_{c2}}+h_{t}$$

If $C_{5}$ firstly occupies conflict point *A*, $t_{c}$ can be expressed as Equation (5):

(5) $$t_{c}=\frac{l_{c5}}{v_{c5}}+h_{t}$$

where $v_{c2}$ is the speed of $C_{2}$, $v_{c5}$ is the speed of $C_{5}$, $l_{c2}$ is the length of $C_{2}$, $l_{c5}$ is the length of $C_{5}$, and $h_{t}$ is the minimum headway.

#### 3.1.4. Definition of Risk Degree

*R* (risk degree) is defined as the probability of conflict or collision occurring between the AV and the surrounding AVs while they remain in their current states. The calculation of the risk degree involves a comparison between the PET t and CPOT $t_{c}$, as expressed by Equation (6).

(6) $$R=\left\{\begin{array}{c} 0\;\;\;,t>t_{c}\\ 1\;\;\;,t\leq t_{c} \end{array}\right.$$

When *R* equals 0, it signifies that the PET between two AVs is greater than the CPOT. In this scenario, after the first AV has passed the conflict point, the conflicting AV can safely navigate the same conflict point after a certain duration. Consequently, it is determined that an acceptable traffic conflict exists, allowing for the AVs to safely traverse the intersection at their maximum speeds.

Conversely, when *R* equals 1, it indicates that the PET between two AVs is either less than or equal to the CPOT. In such instances, it suggests that the conflicting AV is unable to safely pass through the conflict point. Therefore, an unacceptable conflict is recognized, necessitating conflict resolution measures in the subsequent part of the analysis.

### 3.2. Motion Optimization Method

The motion optimization method between two AVs is established by assessing the conflict severity using the previously introduced risk degree.

In cases where *R* equals 0, signifying an acceptable conflict, the AVs can proceed through the intersection at their optimal speeds. The time needed to reach the conflict point is denoted by Equation (7):

(7) $$t_{0}=\left\{\begin{array}{c} \frac{l_{ij1}}{v_{0}},v_{0}=v_{m}\\ \frac{\sqrt{2a_{m}l_{ij1}+{v_{0}}^{2}}-v_{0}}{a_{m}},l_{ij1}<\frac{{v_{m}}^{2}{{-v}_{0}}^{2}}{2a_{m}}\\ \frac{l_{ij1-}\frac{{v_{m}}^{2}{{-v}_{0}}^{2}}{2a_{m}}}{v_{m}}+\frac{v_{m}-v_{0}}{a_{m}},l_{ij1}\geq \frac{{v_{m}}^{2}{{-v}_{0}}^{2}}{2a_{m}} \end{array}\right.$$

where $v_{m}$ is the maximum speed, $l_{ij1}$ is the distance of AV j from the stop line to the conflict point i, $a_{m}$ is the maximum acceleration, and $v_{0}$ is the initial speed of the AV.

In situations where *R* equals 1, indicating an unacceptable conflict, the AVs must decelerate. The DR is defined by Equation (8):

(8) $$a_{d}=\left\{\begin{array}{c} \frac{2\left(l_{ij1}-v_{0}t_{c}\right)}{{t_{c}}^{2}},\;\frac{2\left(l_{ij1}-v_{0}t_{c}\right)}{{t_{c}}^{2}}\leq a_{max}\\ a_{max},\;\frac{2\left(l_{ij1}-v_{0}t_{c}\right)}{{t_{c}}^{2}}>a_{max} \end{array}\right.$$

The additional travel time incurred by the AV due to deceleration is equal to $t_{c}$. If the time needed for deceleration is excessively long, the AV should come to a stop and wait at the stop line.

The time interval from the AV’s arrival at the conflict point to its exit from the intersection can be represented as Equation (9):

(9) $$t_{1}=\left\{\begin{array}{c} \frac{l_{ij2}}{v_{c}},v_{c}=v_{m}\\ \sqrt{\frac{2l_{ij2}}{a_{m}}},l_{ij2}<\frac{{v_{m}}^{2}{{-v}_{c}}^{2}}{2a_{m}}\\ \frac{l_{ij2}}{v_{m}}+\frac{v_{m}}{2a_{m}},l_{ij2}\geq \frac{{v_{m}}^{2}{{-v}_{c}}^{2}}{2a_{m}} \end{array}\right.$$

where $l_{ij2}$ is the distance from the conflict point i to the exit stop line and $v_{c}$ is the next-moment speed of the AV passing the conflict point.

The total time of AV j through the intersection can be shown as follows:

(10) $$t_{j}=\left\{\begin{array}{c} t_{0}+t_{1},t_{0}>t_{c}\\ t_{c}+t_{1},t_{0}\leq t_{c} \end{array}\right.$$

The motion optimization method serves to enhance the AV motion parameters, thereby improving the efficiency and preventing collisions. The cooperative motion optimization involving multiple AVs at intersections builds upon the principles outlined in this section, and the specific formulation method is elucidated in the subsequent section.

## 4. Model Establishment

Numerous studies have employed decision algorithms [25], speed control mechanisms for AVs [26], PID control [27], and fuzzy logic control [28] to govern the movement of AVs within intersections. However, these studies often lack a detailed explanation of how these control strategies can enhance the average travel time and capacity of intersections.

In this section, we establish an AV cooperative motion optimization strategy specifically designed for scenarios involving multi exit lane selection within signalized intersections. This innovative strategy incorporates conflict resolution techniques based on the risk degree, safety car-following principles, and signal schema as constraints. The subsequent sections will provide a comprehensive introduction to these constraints.

### 4.1. Constraints of the Cooperative Motion Optimization Strategy

#### 4.1.1. Safety Car-Following

The minimum safety headway $h_{t}$ between the two AVs is ensured to avoid collision.

(11) $$h_{t}=\frac{l+d_{min}}{v}$$

where l is the AV length, $d_{min}$ is the minimum headway, and v is the speed when the AV reaches the conflict point.

#### 4.1.2. Signal Schema

The intersection signals considered in this paper comprise only two phases: prioritizing east–west traffic initially and subsequently accommodating north–south traffic. It is important to note that the signal control does not extend to right-turn traffic.

#### 4.1.3. Conflict Resolution

AVs are regulated by the conflict resolution scheme based on the risk degree, as detailed in Section 3. It is crucial to understand that the conflict resolution scheme does not alter the chosen driving trajectory route of the AVs but rather adjusts their speed and acceleration while following the designated route.

### 4.2. Operation of the Cooperative Motion Optimization Strategy

**Figure 5:**
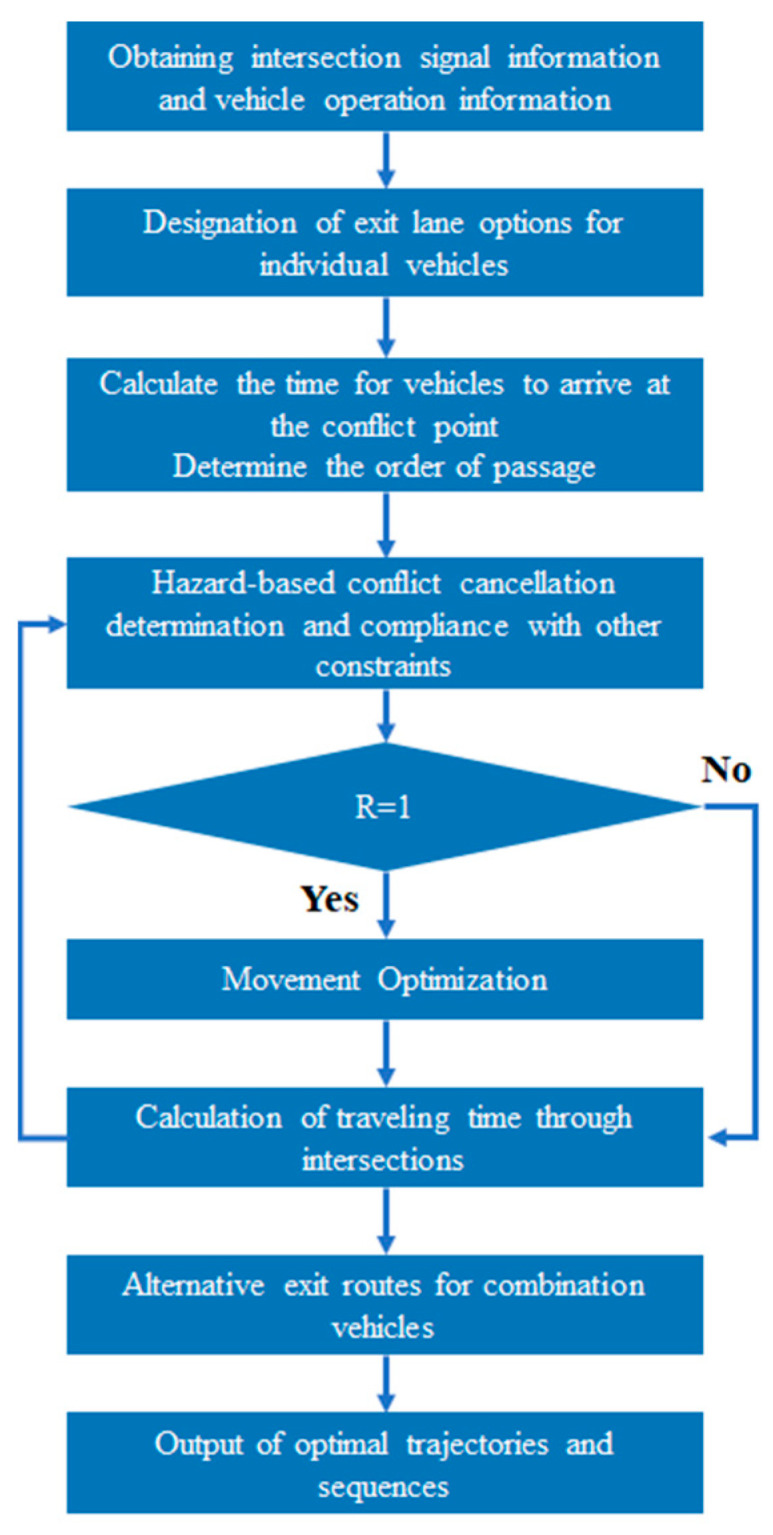
Cooperative process.

**Figure 6:**
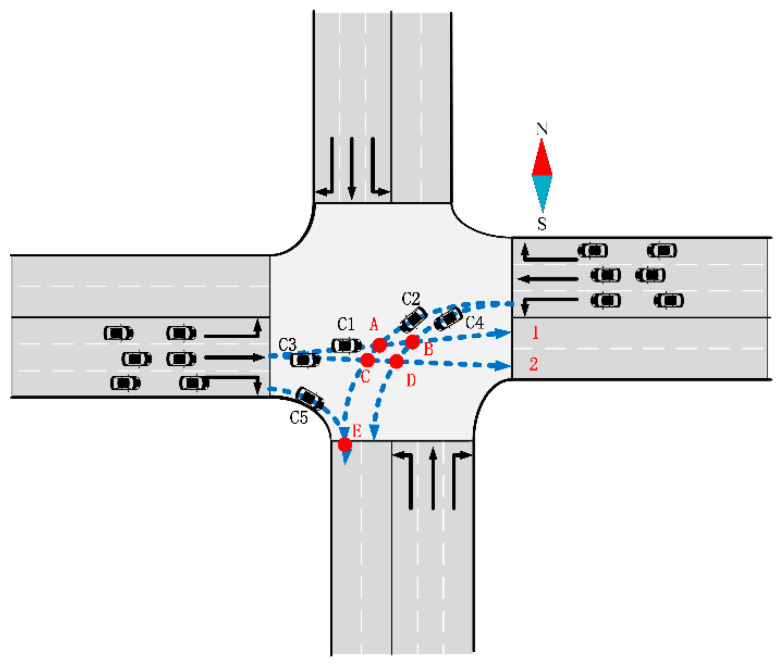
AV cooperative motion optimization strategy.

The cooperative process at the intersection entails AVs efficiently traversing the intersection while prioritizing conflict avoidance. Under traditional intersection control strategies, AVs are confined to fixed exit lanes, with the FCFS control approach being widely employed. This means that the AV reaching the stop line first is given precedence for passage. However, if the first AV progresses slowly toward the conflict point, it can lead to delays for other AVs. Consequently, optimization is necessary for both the trajectories and the sequencing of traffic at the intersection. The specific cooperative process is shown below in Figure 5:

The trajectories and passage sequences of AVs are dynamically adjusted in accordance with the cooperative motion optimization strategy. This strategy ensures that AVs do not come to a halt within the intersection and maximizes the number of AVs that can pass within the green light duration. Figure 6 provides a detailed illustration of the AV cooperative motion optimization strategy.

#### 4.2.1. Exit Lane Selection Combinations

It is assumed that AVs on the section approach at identical speeds and maintain consistent headway spacing. Additionally, AVs possess the capability to exchange information with one another. Each AV is presented with multiple exit lane options, leading to the construction of a trajectory set that includes all available choices.

For instance, AVs *C1*, *C2*, *C3*, *C4*, and other AVs each have two exit lane choices; *C5* and other right-turn AVs only have one choice. To illustrate, we can consider one of the exit lane choices, as depicted in Figure 6, where *C1* selects exit lane 1, and *C2* opts for exit lane 2.

#### 4.2.2. Trajectory Control

The timing is determined when an AV reaches a known conflict point position, aligning with the assumption stated in Section 4.2.1. Subsequently, the time at which conflicting AVs, such as *C1* and *C2*, reach conflict point A can be calculated. This process allows for ranking the arrival times of all AVs at their respective conflict points. The AV that reaches the corresponding conflict point first is granted priority for passage.

Distinct traffic sequences lead to varied traffic strategies, including speed and acceleration regulation. AVs with priority are permitted to travel at maximum speed if their current speed is already at the maximum limit. However, if an AV has not yet reached the maximum speed, it can accelerate to attain the optimal speed within the limits of maximum acceleration, thus facilitating passage through the intersection.

For AVs in conflict with those holding the right of way, they must adhere to speed and acceleration controls in accordance with the conflict resolution scheme based on the risk degree. Following the passage of the conflict point, they can also accelerate using maximum acceleration while moving through the intersection.

#### 4.2.3. Obtain the Optimal Path and the Optimal Traffic Order

Once an AV has successfully traversed the conflict point and its trajectory no longer affects other AVs within the intersection, it can be excluded from the decision set and proceed to make cooperative traffic decisions for other AVs. This process entails specifying the exit lane selections for various combinations of AVs and continuing with the cooperative strategy as described in Section 4.2.2. By following this approach, the traffic time for different combinations of exit lanes for all AVs is obtained. Subsequently, a comparison is made to determine the optimal traffic trajectory combination.

## 5. Experiments and Analysis

This paper’s cooperative motion optimization strategy is validated through simulation in two distinct traffic scenarios. A comparative analysis is conducted by contrasting this strategy with the FCFS approach. These scenarios include uneven traffic flow at the entrance and a complete signal cycle, serving as representative examples for evaluation.

### 5.1. Basic Parameters of the Scenario

Each AV is configured with a maximum speed of 40 km/h and a maximum acceleration/deceleration capability of 2.5 m/s^2^. The initial speeds for through and left-turn AVs are set at 30 km/h, while right-turn AVs start at 25 km/h. To ensure safety, a minimum headway of 0.5 s is enforced for all the AVs, with a minimum AV spacing of 2 m.

**Figure 7:**
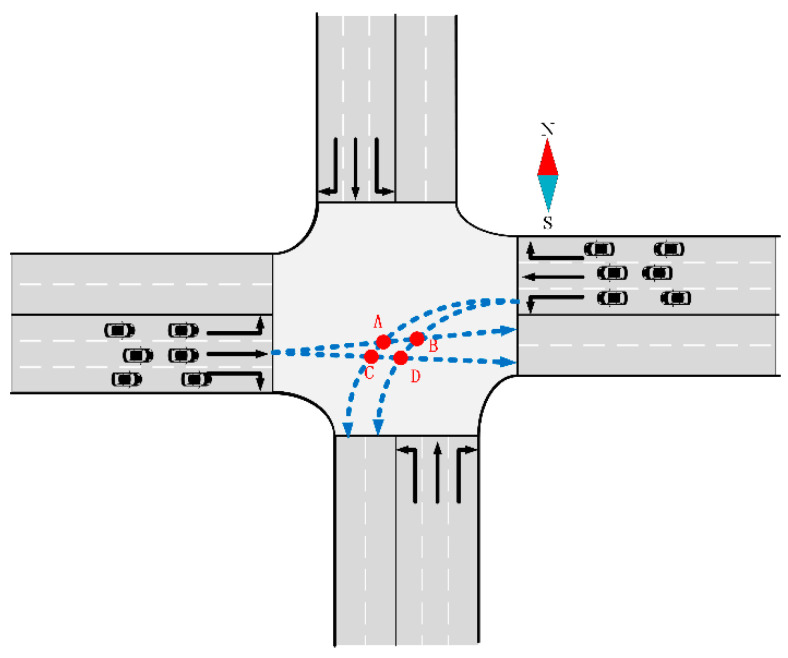
Plane intersection conflict point location diagram.

The entrance lane width is designated as 3 m, and the exit lane width is 3.5 m. For reference, Figure 7 and Table 1 provide specific distances from the stop line to the conflict points, using the west through entrance and east left-turn entrance as illustrations. These conflict points for the west entrance and the east left-turn entrance serve as representative locations for all the cross-conflicts within the intersection.

In the case of crossing conflicts, the default conflict distances specified in Table 1 are employed. Additionally, the merging conflict distance equals the distance covered by right-turn AVs or through AVs as they travel from the stop line to the exit lane.

### 5.2. Simulation Experiments

#### 5.2.1. Scenario 1

**Figure 8:**
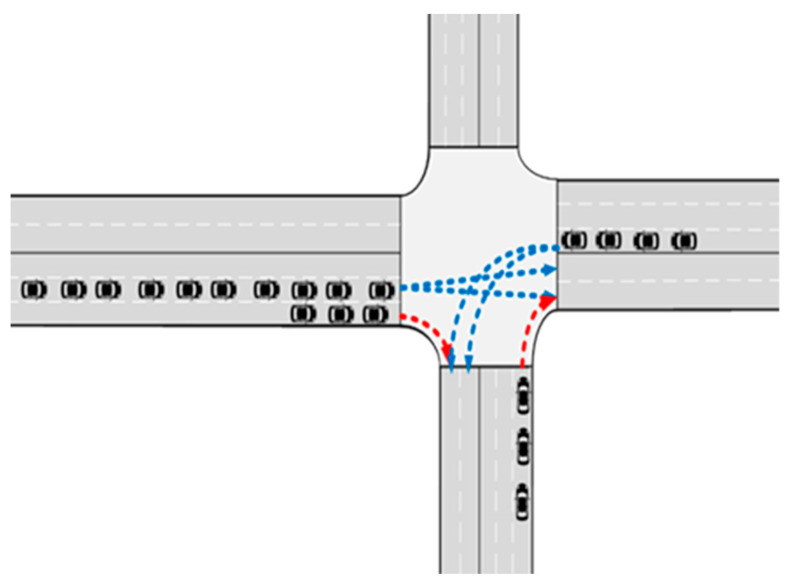
Simulation scenario 1.

In Figure 8, there are a total of 10 through AVs arriving at the west entrance, 4 left-turn AVs arriving at the east entrance, and 3 right-turn AVs individually approaching the west entrance and south entrance.

The simulation results are shown in Table 2.

The implementation of the strategy outlined in this paper yields improvements in various metrics. Specifically, the mean travel time, mean number of halts, and mean speed of the AVs all exhibit enhancements. Most notably, there is a substantial improvement in the mean time loss, which registers a significant increase of 42.66%.

#### 5.2.2. Scenario 2

**Figure 9:**
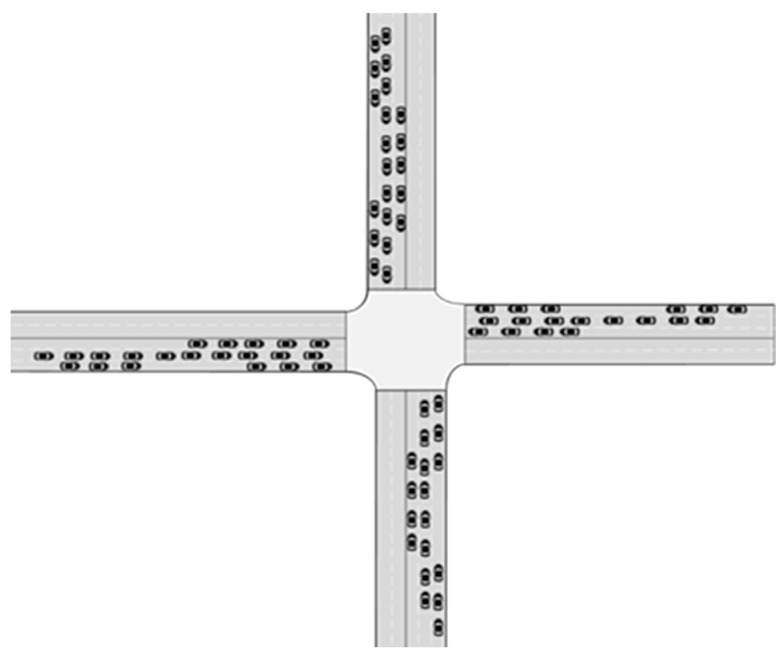
Simulation scenario 2.

In Figure 9, the AVs at all the entrances arrive at the stop line in a randomized manner. The signal timing scheme is established with consideration for the green time, yellow time, and red time. It is important to note that right-turn traffic is not subject to signal control in China and can proceed freely.

The signal schema at the intersection unfolds as follows: the east–west traffic is granted passage during the initial phase of the signal for a duration of 33 s, followed by a 3 s yellow time and a subsequent 3 s red time. Subsequently, the north–south traffic is given the right of way for a duration of 33 s.

The implementation of this paper’s strategy results in improvements across multiple metrics for the AVs. Specifically, in Table 3, there are enhancements in the mean travel time, mean time loss, mean number of halts, and mean speed. It is worth noting that while the efficiency gains achieved are not exceptionally large, this is partly attributable to the effect of the signal timing, especially the queuing time for the AVs awaiting another phase, which can influence the overall efficiency.

### 5.3. Result Analysis

We examine scenario 2 to conduct a detailed analysis of the spatiotemporal trajectories and acceleration patterns of the AVs, as governed by the control scheme outlined in this paper. The spatiotemporal maps illustrating the AV trajectories in each of the four directions are presented below.

#### 5.3.1. Spatiotemporal Trajectory

The trajectories of the AVs following the cooperative strategy outlined in this paper are depicted in yellow, while the AVs not subject to the control scheme of this paper are represented in blue. It can be seen from the figure that the yellow line segments, i.e., the vehicles using the control method in this paper, are able to pass through the intersection with faster passing efficiency.

**Figure 10:**
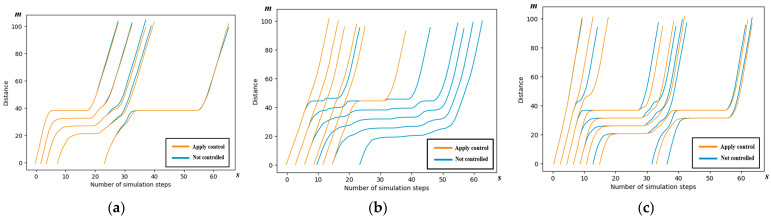
Spatiotemporal trajectory comparison of the east entrance in simulation scenario 2. (**a**) Spatiotemporal trajectory comparison of left-turn AVs; (**b**) spatiotemporal trajectory comparison of right-turn AVs; and (**c**) spatiotemporal trajectory comparison of through AVs.

Observations from Figure 10 and Table 4 reveal significant enhancements in the traffic efficiency of all the right-turn AVs. Additionally, some of the through AVs experience improved traffic efficiency. However, it is noteworthy that the traffic efficiency of the left-turn AVs remains largely unchanged compared to the AVs without control.

**Figure 11:**
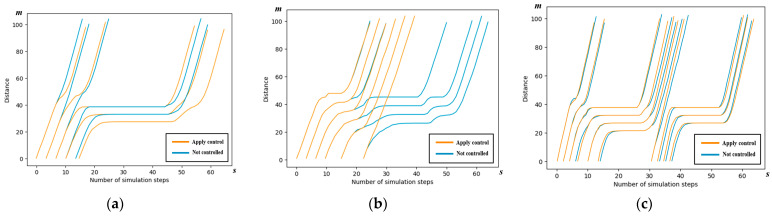
Spatiotemporal trajectory comparison of the west entrance in simulation scenario 2. (**a**) Spatiotemporal trajectory comparison of left-turn AVs; (**b**) spatiotemporal trajectory comparison of right-turn AVs; and (**c**) spatiotemporal trajectory comparison of through Avs.

In Figure 11, the traffic efficiency of both the left-turn and through AVs is decreased. Nevertheless, an intriguing observation from Table 5 is that the traffic efficiency of all directions is lower than that of those without control. This phenomenon is a result of some left-turn and through AVs slowing down deliberately to ensure overall traffic efficiency and avoid collisions. 

**Figure 12:**
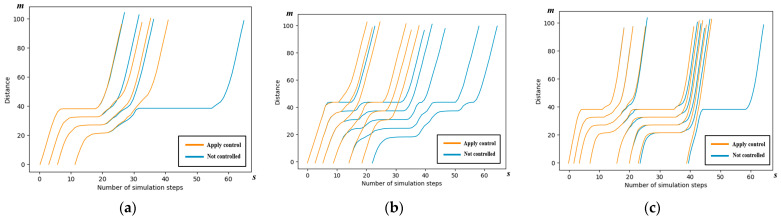
Spatiotemporal trajectory comparison of the south entrance in simulation scenario 2. (**a**) Spatiotemporal trajectory comparison of left-turn AVs; (**b**) spatiotemporal trajectory comparison of right-turn AVs; and (**c**) spatiotemporal trajectory comparison of through Avs.

In Figure 12, similar efficiency improvements are observed at the south entrance. However, it is important to note that the improvements in traffic efficiency and the reduction in time loss, as indicated in Table 6, are not notably pronounced. This is primarily because the north–south AVs must wait for a duration of 33 s while the east–west AVs pass. Consequently, the decrease in the average speed and the parking rate can be attributed to this waiting period.

**Figure 13:**
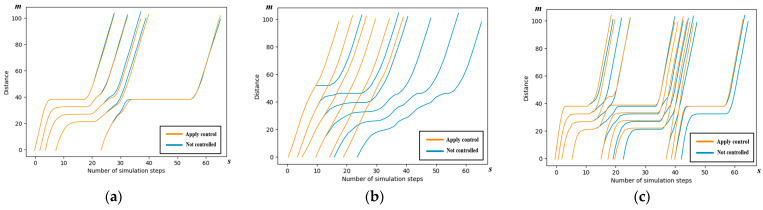
Spatiotemporal trajectory comparison of the north entrance in simulation scenario 2. (**a**) Spatiotemporal trajectory comparison of left-turn AVs; (**b**) spatiotemporal trajectory comparison of right-turn AVs; and (**c**) spatiotemporal trajectory comparison of through Avs.

In Figure 13, while several other metrics have exhibited significant improvements, it is notable that the parking rate experiences a reduction, as indicated in Table 7. This decline is primarily attributed to the AVs continuing to approach the intersection and parking in front of the stop line during the waiting period for the green light.

The cooperative motion optimization method proposed in this paper has been effectively validated for its optimization effect on the spatiotemporal traffic efficiency of AVs across all directions. It is worth emphasizing that ensuring the stability and efficiency of AV traffic through acceleration remains a crucial aspect of this research.

#### 5.3.2. Analysis of AV Acceleration

**Figure 14:**
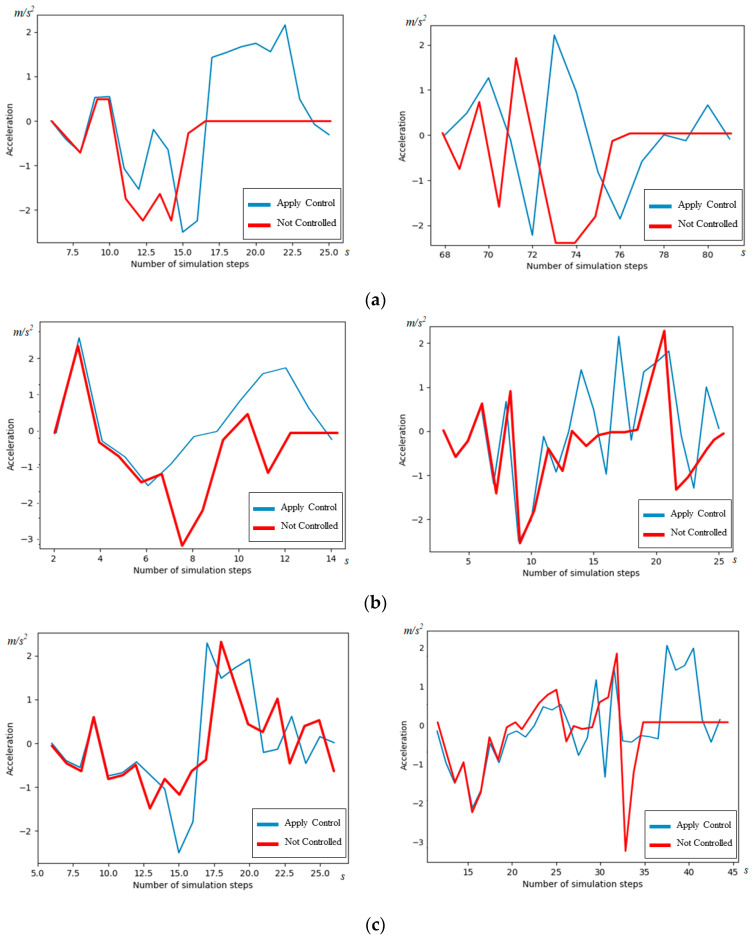
Comparison of AV acceleration in simulation scenario 2. (**a**) Two representative straight-through AVs; (**b**) two representative right-turn AVs; and (**c**) two representative left-turn AVs.

The changes in acceleration for the representative AVs are visualized in Figure 14 and Table 8. The AVs’ acceleration patterns when applying the cooperative strategy outlined in this paper are represented in blue, while those using the comparison method are shown in red. It can be found that the overall rate of sharp change in acceleration, the method in this paper, has smoother acceleration than the original method, resulting in a reduction in the rate of sharp change in acceleration by up to 38.1%.

In Figure 14a, the red curve remains at 0 in the second half, indicating that the AV is parked and causing a decrease in traffic efficiency. In contrast, in Figure 14b,c, the blue curve displays significantly less variability compared to the red curve, illustrating that the cooperative strategy presented in this paper effectively and smoothly regulates the acceleration of AVs.

It is worth noting that AVs not subject to the cooperative strategy in this paper occasionally experience maximum deceleration, suggesting that the strategy outlined in this paper enhances the stability of AV traffic.

## 6. Conclusions

To address the challenges of underutilized spatial and temporal resources and the subjective nature of conflict severity indicators in intersection efficiency, this paper has introduced a cooperative motion optimization method based on risk degree.

The flexibility of exit lane choices expands spatial resources and forms a fundamental condition for optimizing AV movements. The primary focus of the motion optimization method lies in the assessment of the risk degree. The risk degree avoids the artificial imposition of thresholds for conflict severity by directly comparing two indicators, thus providing a more objective measure. The conflict motion optimization method has been formulated to enhance the motion dynamics between any two AVs, serving as the theoretical basis for the intersection cooperative motion optimization strategy presented in this paper. This approach extends to the optimization of overall intersection motion when multiple AVs traverse it, considering it not as a mere superposition of individual motion optimization methods but as a comprehensive approach that accounts for the set of conflicting AVs.

Through SUMO-based numerical simulations and a comparison with FCFS, the cooperative motion optimization strategy outlined in this paper has been verified. In the two scenarios, it resulted in efficiency improvements of 20.51% and 11.59%, increased traffic speeds by 18.38% and 5.92%, and reduced parking rates by 14.47% and 5.32%, respectively. Furthermore, the analysis of the acceleration patterns after simulation demonstrates that the cooperative strategy in this paper leads to smoother AV movements. These results confirm the effectiveness and stability of the intersection cooperative strategy in an autonomous driving environment.

The method in this paper does not take into account the complex traffic environment of pedestrians, non-motorized vehicles, more complex vehicle control methods, etc. To better improve the generalizability and efficiency of the motion optimization method, in the future, this research aims to expand in two key areas: the precise control of AV motion parameters to achieve enhanced stability and the development of a unified framework for expressing AV trajectory routes under different intersection layouts, making the cooperative motion optimization strategy more adaptable to real-world scenarios.

## Figures and Tables

**Table 1:** Distance from the stop line to the conflict point.

Distance from Stop Line to Conflict Point		
Conflict Point	Go Straight on West Entrance Road	Turn Left on East Entrance Road
	Distance/m	Distance/m
A	8.5	10.3
B	11.1	12.8
C	9.8	13.2
D	12.7	14.5

**Table 2:** Comparison of cooperative traffic schemes in scenario 1.

	Not Controlled	Apply Control	Optimization Rate
Mean Travel Time/s	15.75	12.52	20.51%
Mean Time Loss/s	7.22	4.14	42.66%
Mean Halts Per AV/s	0.76	0.65	14.47%
Mean Speed/m/s	4.95	5.86	18.38%

**Table 3:** Comparison of cooperative traffic schemes in scenario 2.

	Not Controlled	Apply Control	Optimization Rate
Mean Travel Time/s	34.81	30.79	11.59%
Mean Time Loss/s	27.24	23.30	14.47%
Mean Halts Per AV/s	0.94	0.89	5.32%
Mean Speed/m/s	3.21	3.40	5.92%

**Table 4:** Comparison of cooperative traffic schemes of the east entrance.

	Not Controlled	Apply Control	Optimization Rate
Mean Travel Time/s	26.03	17.77	31.73%
Mean Time Loss/s	18.11	9.89	45.39%
Mean Halts Per AV/s	1.00	0.50	50.00%
Mean Speed/m/s	3.92	5.24	33.67%

**Table 5:** A comparison of the cooperative traffic schemes of the west entrance.

	Not Controlled	Apply Control	Optimization Rate
Mean Travel Time/s	20.96	23.99	−14.46%
Mean Time Loss/s	12.70	15.80	−24.41%
Mean Halts Per AV/s	0.78	0.89	−14.10%
Mean Speed/m/s	4.61	3.97	−13.88%

**Table 6:** A comparison of the cooperative traffic schemes of the south entrance.

	Not Controlled	Apply Control	Optimization Rate
Mean Travel Time/s	40.45	38.50	4.82%
Mean Time Loss/s	32.67	30.81	5.69%
Mean Halts Per AV/s	0.89	0.94	−5.62%
Mean Speed/m/s	2.53	2.41	−4.74%

**Table 7:** Comparison of cooperative traffic schemes of the north entrance.

	Not Controlled	Apply Control	Optimization Rate
Mean Travel Time/s	51.7	42.90	17.02%
Mean Time Loss/s	45.47	36.70	19.29%
Mean Halts Per AV/s	1.11	1.22	−9.91%
Mean Speed/m/s	1.76	1.98	12.50%

**Table 8:** Comparison of accelerations.

	(a)	(b)	(c)
Max acceleration difference of our method	4.5	3.7	2.9
Max acceleration difference of comparison method	4.2	5.2	4.7
Rate of change	7%	−28.8%	−38.1%

## Data Availability

All the data included in this study are available upon request by contact with the corresponding author.
